# Early Anti-Rhabdomyolysis Infusion Therapy Before Tourniquet Release Is Associated with Reduced Acute Kidney Injury, Limb Amputation, and Mortality in Combat-Related Lower Extremity Injuries: A Retrospective Cohort Study

**DOI:** 10.3390/jcm15062123

**Published:** 2026-03-11

**Authors:** Vitalii A. Lukiianchuk, Wojciech Barg, Oleksandr V. Oliynyk, Svitlana M. Yaroslavska, Arsen A. Gudyma, Tomasz Jurek

**Affiliations:** 1 Zaporizhzhya Military Hospital, 69063 Zaporizhzhya, Ukraine; lukianchuk.v@i.ua; 2 Department of Anesthesiology and Intensive Care, Bogomolets National Medical University, 01601 Kyiv, Ukraine; 3 Department of Health Promotion, Faculty of Physical Culture Sciences, Medical College, University of Rzeszów, 35-025 Rzeszów, Poland; 4 Department of Emergency Medicine, Ternopil National Medical University, 46000 Ternopil, Ukraine; 5 Battlefield Medicine and Forensic Ballistics Laboratory, Department of Forensic Medicine, Faculty of Medicine, Wroclaw Medical University, Jana Mikulicza-Radeckiego 4, 50-345 Wroclaw, Poland

**Keywords:** combat trauma, tourniquet, rhabdomyolysis, acute kidney injury, ischemia–reperfusion injury, infusion therapy, military medicine

## Abstract

**Background**: Combat-related lower extremity injuries frequently require prolonged tourniquet application to control life-threatening hemorrhage. Although effective for hemorrhage control, prolonged ischemia followed by reperfusion substantially increases the risk of rhabdomyolysis, acute kidney injury (AKI), limb loss, and mortality. The optimal timing of anti-rhabdomyolysis infusion therapy in relation to tourniquet release remains uncertain. **Methods**: This retrospective single-center cohort study analyzed 120 Ukrainian military casualties with combat-related lower extremity injuries requiring prolonged tourniquet application and subsequent surgical management, including fasciotomy and tourniquet release. Patients were divided into two groups based on infusion strategy: standard therapy initiated after tourniquet release and early anti-rhabdomyolysis infusion therapy initiated before tourniquet removal during the ischemic phase. Primary outcomes included dialysis-requiring AKI, limb amputation, and death. Multivariable logistic regression models were adjusted for baseline physiological severity, including shock index at admission and baseline acid–base status. Model performance was evaluated using the Akaike Information Criterion (AIC) and receiver operating characteristic (ROC) analysis. Propensity score–based inverse probability of treatment weighting (IPTW) was applied as a sensitivity analysis. **Results**: After adjustment, early infusion therapy was independently associated with lower rates of dialysis-requiring AKI (adjusted odds ratio [OR] 0.33; 95% confidence interval [CI] 0.13–0.84; *p* = 0.020), limb amputation (OR 0.32; 95% CI 0.11–0.95; *p* = 0.040), and mortality (OR 0.23; 95% CI 0.07–0.77; *p* = 0.017). Adjusted models demonstrated good discriminative ability, with areas under the ROC curve of 0.813 for AKI, 0.838 for amputation, and 0.823 for mortality. Sensitivity analyses using IPTW yielded consistent results. **Conclusions**: In combat-related lower extremity injuries requiring prolonged tourniquet application, early initiation of anti-rhabdomyolysis infusion therapy prior to reperfusion is associated with significantly reduced risks of severe AKI, limb loss, and death. These findings suggest that preventive renal-protective strategies initiated before tourniquet release may improve outcomes in high-risk military trauma settings and warrant further prospective investigation.

## 1. Introduction

In present-day military conflicts, extremity combat wounds (ECW) represent the most common type of injury, largely due to the widespread use of personal protective equipment that reduces fatal torso trauma. Musculoskeletal injuries account for approximately 50% of all combat wounds, with explosive mechanisms responsible for more than 75% of casualties reported during military operations in Iraq and Afghanistan [1,2]. In a large cohort comprising 3575 ECW from these conflicts, penetrating soft-tissue injuries constituted 53% of cases, while fractures accounted for 26%, of which 82% were open fractures. Fractures were equally distributed between the upper and lower limbs, with nearly half of lower extremity fractures involving the tibia and fibula [2].

Similar patterns have been observed in the ongoing armed conflict in Ukraine. According to recent reports, approximately 40% of combat injuries involve fractures of long tubular bones, 30% affect the soft tissues of the extremities, and the remaining 30% involve other anatomical regions. Among casualties with extremity injuries, approximately 10% experience massive hemorrhage requiring urgent hemostatic intervention [3]. A recent retrospective study analyzing data from 2014 to 2022 and including 2496 casualties with limb injuries requiring tourniquet application reported lower extremity involvement in 84.4% of cases. Tourniquet application time (TAT) ranged from 50 to 380 min, with a mean duration of 205.9 min. Notably, reported mortality and limb amputation rates were relatively low at 4.0% and 3.7%, respectively [4].

Critical bleeding in ECW is defined as rapid blood loss leading to progressive and potentially fatal hypovolemic shock. Such bleeding most commonly results from injury to major arterial vessels or complex multiple vascular damage. Immediate and effective hemorrhage control is therefore essential. For example, a casualty with femoral artery injury may lose consciousness within one minute and die shortly thereafter if effective hemostasis is not achieved [5].

Hemostatic tourniquets represent a reliable and effective method for rapid control of life-threatening extremity hemorrhage. In the Ukrainian Armed Forces, each soldier is equipped with a tourniquet and trained in its application for self-aid or buddy-aid, allowing hemorrhage control to be initiated directly on the battlefield. This practice has significantly reduced immediate combat fatalities. However, prolonged tourniquet application is associated with substantial secondary tissue injury. Experimental and clinical data indicate that irreversible ischemic tissue damage may begin approximately three hours after complete interruption of blood flow [6].

According to established military medical procedures, tourniquet conversion or temporary loosening is recommended every 2–3 h to mitigate ischemic injury when feasible. Information regarding tourniquet placement time and any conversion attempts is documented on a casualty card accompanying the wounded soldier throughout evacuation and treatment. During the study period, however, tourniquet manipulation was permitted exclusively for physicians, particularly in cases of prolonged TAT. As a result, recommended periodic tourniquet conversion was frequently not performed due to delayed evacuation, limited availability of medical personnel, and ongoing combat conditions. Consequently, continuous tourniquet application often persisted for several hours and in some cases exceeded six hours.

Prolonged limb ischemia results in critical disturbances of oxygen delivery and metabolic waste elimination, triggering inflammatory cascades and cellular injury. Severe hypoperfusion with hypoxia and hypercapnia leads to increased concentrations of vasoactive mediators, including histamine, serotonin, and tumor necrosis factor, with subsequent endothelial dysfunction and increased vascular permeability [7]. Fluid shifts into the interstitium cause tissue edema, while hypoxia, oxidative stress, and metabolic derangements disrupt cellular osmotic balance. These processes culminate in cellular swelling, membrane dysfunction, necrosis, and increased intracompartmental pressure, ultimately leading to acute extremity compartment syndrome (AECS) [8].

In combat settings, additional factors may exacerbate tissue injury. Although soldiers are trained in tourniquet application, they are not medical professionals, and improper application is common. In many cases, insufficient arterial occlusion with preserved venous inflow occurs, resulting in venous congestion and increased intrafascial pressure [9]. Combined with external compression commonly used in combat wound management, these factors further promote AECS, muscle necrosis, and the development of rhabdomyolysis (RM). This initiates a vicious cycle leading to acute kidney injury (AKI), limb amputation, and potentially death.

Timely fasciotomy reduces intracompartmental pressure, improves tissue perfusion, facilitates drainage of toxic edema fluid, and mitigates ischemic injury. According to current regulations in the Ukrainian Armed Forces, fasciotomy is performed upon first contact with qualified surgical personnel, typically at advanced surgical units (ASU). Tourniquet removal is subsequently performed, resulting in limb reperfusion.

Reperfusion of ischemic muscle leads to systemic release of muscle breakdown products, including myoglobin, potassium, phosphate, and inflammatory mediators. This process may result in metabolic acidosis, electrolyte disturbances, myoglobinemia, myoglobinuria, and multiple organ dysfunction, with acute kidney injury representing the most clinically significant complication [1,10]. Rhabdomyolysis has been reported in approximately 25–30% of wounded soldiers during recent military operations, with injured soldiers being three to four times more likely to develop RM than civilian trauma patients [11,12]. Identified risk factors include extremity injury, explosive mechanisms, high injury severity scores, shock index greater than 0.9, and delayed evacuation. Importantly, the presence of RM more than doubles the risk of AKI [12].

Free circulating myoglobin is the principal pathological mediator of RM-induced AKI. Its nephrotoxic effects include direct tubular cytotoxicity, intratubular cast formation with uromodulin, oxidative injury mediated by free iron, and renal vasoconstriction [2,7,8]. Prevention of AKI therefore remains the cornerstone of RM management and is primarily achieved through early restoration of intravascular volume, maintenance of renal perfusion, and dilution of intratubular myoglobin. However, the optimal timing for initiating preventive therapy, particularly in the absence of overt clinical signs of RM, remains uncertain [9,10].

In current Ukrainian military practice, intensive infusion therapy—including crystalloids, osmotically active solutions, and urine alkalization—is initiated once clinical signs of RM become evident. Prior to tourniquet removal, however, such signs are typically absent, and clear indications for therapy initiation are lacking. Whether initiating anti-rhabdomyolysis infusion therapy before reperfusion and before the appearance of clinical RM manifestations confers clinical benefit remains an open and clinically relevant question.

Therefore, the aim of this study was to evaluate the impact of early initiation of infusion therapy relative to tourniquet removal on the risk of death, dialysis-requiring acute kidney injury, and limb amputation in casualties with lower extremity combat wounds complicated by rhabdomyolysis.

## 2. Materials and Methods

### 2.1. Study Design and Setting

This retrospective single-center observational study was conducted at a military medical facility providing surgical and intensive care to casualties with combat-related injuries. The study period included patients treated during active hostilities. This study analyzed the medical records of 120 wounded Ukrainian Army soldiers who were treated in medical units of the Zaporizhzhia Military Hospital from 1 June 2023 to 31 August 2023. The assumptions and plan of the research were discussed and approved by the Ethics Committee of the Ternopil National Medical University, Ukraine, on 25 May 2023.

The study analyzed medical records of casualties with lower extremity combat wounds requiring prolonged tourniquet application and subsequent surgical management. Patients were managed according to local clinical protocols under wartime conditions.

### 2.2. Study Population

Eligible patients met the following inclusion criteria:

- combat-related injury to the lower extremity;
- application of a hemostatic tourniquet prior to hospital admission;
- prolonged tourniquet application time (TAT);
- surgical treatment including fasciotomy and tourniquet removal;
- availability of clinical and laboratory data for analysis.

Patients with incomplete medical records or missing key outcome data were excluded from the analysis.

### 2.3. Treatment Strategy

Clinically important combat wounds were defined as injuries that, in the opinion of a military surgeon, could potentially influence the patient’s subsequent clinical course.

Following injury, all casualties received prehospital hemorrhage control with tourniquet application and were transported to stabilization points. Initial management included peripheral venous access, urinary bladder catheterization, administration of analgesics, and intravenous infusion of approximately 1 L of electrolyte solution.

Subsequently, patients were evacuated to an advanced surgical unit (ASU). At the ASU, initial resuscitation typically included transfusion of approximately 500 mL of group O Rh-negative red blood cells and 500 mL of group AB lyophilized plasma. Surgical wound debridement and fasciotomy were performed as indicated.

Allocation to treatment groups was not randomized and did not depend on individual patient characteristics. Instead, it reflected organizational and protocol-based differences in clinical practice between comparable medical units and consecutive time periods during the study interval.

Importantly, the same inclusion criteria, surgical approach, and perioperative monitoring protocols were applied across units. No intentional selection of patients for a specific infusion strategy was performed by treating physicians.

Infusions administered prior to tourniquet removal in both groups were delivered over approximately 30 min.

In the conventional treatment group, patients received an additional 1000 mL of saline solution before tourniquet removal. After reperfusion, infusion therapy included 500 mL of 4% gelatin solution (Gelofusine, B. Braun, Melsungen, Germany), 100 mL of 4% sodium bicarbonate solution (Yuria-Pharm, Kyiv, Ukraine), and 100 mL of 15% mannitol solution (Yuria-Pharm, Kyiv, Ukraine).

In the early anti-rhabdomyolysis treatment group, infusion therapy was initiated before tourniquet removal during the ischemic phase and consisted of 500 mL of 4% gelatin solution, 100 mL of 4% sodium bicarbonate solution, and 100 mL of 15% mannitol solution. After tourniquet removal, patients received 1000 mL of saline solution.

Importantly, baseline demographic characteristics, tourniquet application time, and shock index at admission were largely comparable between groups.

### 2.4. Data Collection

Demographic, clinical, and laboratory data were extracted from medical records. Collected variables included age, tourniquet application time, shock index at admission (SI-1) and after initial resuscitation (SI-2), laboratory markers of rhabdomyolysis and renal function, including urinary hemoglobin/myoglobin and serum creatinine at 24 h (Cr-24).

Shock index (SI) was defined as the ratio of heart rate to systolic blood pressure. It was assessed twice: on arrival at the ASU (SI-1) and immediately before tourniquet removal (SI-2). Accordingly, SI measurements were obtained approximately 35–45 min apart.

Urinary hemoglobin and urinary pH were assessed using a rapid dipstick test (Citolab 10, Pharmasco, Kyiv, Ukraine) at three time points: immediately before tourniquet removal, 30 min after tourniquet removal, and approximately 24 h later. In the combat setting, dipstick-detected urinary hemoglobin was used as a surrogate marker for myoglobinuria. These measurements were denoted as Hb-1, Hb-2, Hb-3 and pH-1, pH-2, pH-3, respectively.

Serum creatinine was measured concurrently with the third urine dipstick test and was referred to as Cr-24.

### 2.5. Outcomes

Approximately two hours after tourniquet removal, patients underwent final evacuation to a military hospital.

The primary endpoint of the study was 28-day mortality. Secondary endpoints included dialysis-requiring acute kidney injury (AKI) within 28 days and irreversible ischemic damage of the injured limb requiring partial or complete amputation within 28 days.

Indications for renal replacement therapy were determined according to local clinical recommendations and included at least one of the following criteria: estimated glomerular filtration rate < 10 mL/min/1.73 m^2^, serum urea > 35 mmol/L, hyperkalemia > 6.5 mmol/L, serum bicarbonate < 20 mmol/L, or cerebral, pulmonary, or pericardial edema refractory to conservative treatment.

### 2.6. Statistical Analysis

Continuous variables were tested for normality using visual inspection and distribution assessment (histograms and Q–Q plots). Statistical analysis was performed using Python (version 3.11, Python Software Foundation, Wilmington, DE, USA) with the statsmodels (version 0.14) and scikit-learn (version 1.3) libraries. Between-group comparisons for continuous variables were performed using the Mann–Whitney U test.

Categorical variables are presented as counts and percentages and were compared using the χ^2^ test or Fisher’s exact test, as appropriate.

Univariable logistic regression was used to estimate crude odds ratios (OR) with 95% confidence intervals (CI) for the association between early infusion therapy and each clinical outcome. Multivariable logistic regression models were constructed to adjust for baseline physiological severity and included treatment group, shock index at admission, and baseline urinary pH.

Model performance was assessed using the Akaike Information Criterion (AIC), with lower values indicating improved model fit. Discriminative ability was evaluated using receiver operating characteristic (ROC) curves and the area under the curve (AUC).

As a sensitivity analysis to address potential confounding by indication, propensity scores were estimated using baseline covariates (age, tourniquet application time, shock index at admission, baseline urine pH, and baseline urinary hemoglobin). Stabilized inverse probability of treatment weights (IPTW) were applied to achieve covariate balance (standardized mean differences < 0.10). Weighted logistic regression models were then fitted to estimate the association between early infusion therapy and each outcome.

All statistical tests were two-sided, and a *p*-value < 0.05 was considered statistically significant.

Artificial intelligence-based tools were used exclusively for language editing and stylistic improvement of the manuscript. No AI tools were used for data analysis, interpretation of results, or generation of scientific content.

## 3. Results

### 3.1. Baseline Characteristics

Baseline demographic and clinical characteristics of the study groups are presented in Table 1. The groups were comparable in terms of age, duration of tourniquet application (TAT), and shock index at admission (SI-1), indicating a similar initial severity of injury. No statistically significant differences were observed in baseline urinary pH (pH-1). Although most baseline characteristics were comparable between groups, baseline urinary hemoglobin (Hb-1) was slightly higher in the standard treatment group.

After initiation of treatment and prior to reperfusion, patients receiving early anti-rhabdomyolysis infusion therapy demonstrated significantly lower shock index values after initial resuscitation (SI-2) compared with the standard treatment group (*p* < 0.001). Laboratory dynamics revealed significantly higher urinary hemoglobin levels at 30 min (Hb-2) in the early-treatment group (*p* = 0.027), while no significant differences were observed at 24 h (Hb-3). Importantly, serum creatinine measured 24 h after admission (Cr-24) was significantly lower in the early-treatment group (*p* = 0.007).

### 3.2. Clinical Outcome Analysis 

Clinical outcomes are summarized in Table 2. Patients treated with the early infusion protocol had lower rates of dialysis-requiring acute kidney injury, limb amputation, and death compared with those receiving standard treatment.

### 3.3. Logistic Regression Analysis

Results of univariable and multivariable logistic regression analyses are presented in Table 3. In crude (unadjusted) models, early anti-rhabdomyolysis infusion therapy was not significantly associated with dialysis or limb amputation, while a non-significant trend toward reduced mortality was observed.

After adjustment for baseline physiological severity, including shock index at admission and baseline acid–base status, early infusion therapy emerged as an independent protective factor for all studied outcomes. Adjusted analysis demonstrated a significant reduction in the odds of dialysis, amputation, and death compared with standard treatment. The reported AUC values reflect the discriminative performance of the full adjusted multivariable logistic regression models, which included treatment group, shock index at admission, and baseline acid–base status, rather than the predictive ability of early infusion therapy alone.

Model performance improved after adjustment, as reflected by lower Akaike Information Criterion (AIC) values across all outcomes, indicating better model fit.

As a sensitivity analysis to address potential confounding by indication, propensity score–based inverse probability of treatment weighting (IPTW) was applied using baseline covariates. The IPTW analysis yielded effect estimates consistent with the primary multivariable models, supporting the robustness of the observed associations.

### 3.4. Model Discrimination

**Figure 1:**
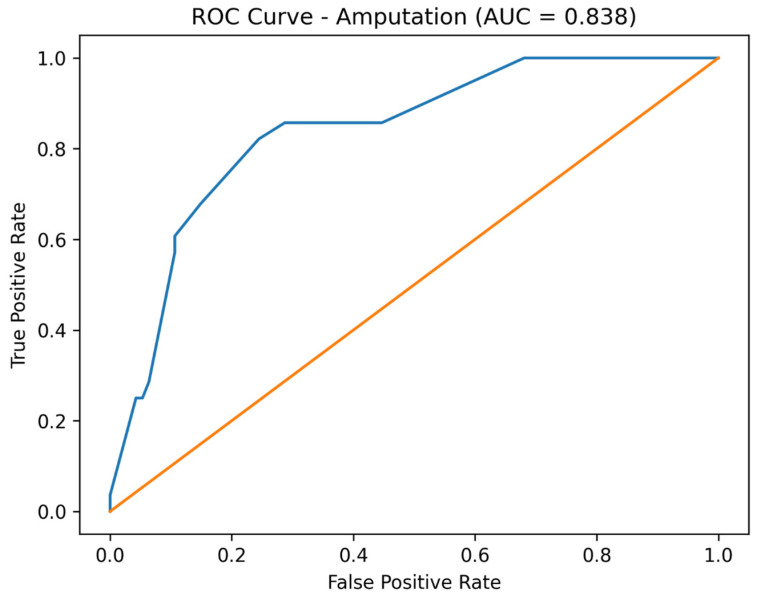
Receiver operating characteristic (ROC) curve for prediction of amputation using the adjusted logistic regression model (AUC = 0.838). The orange line represents the ROC curve of the adjusted model.

**Figure 2:**
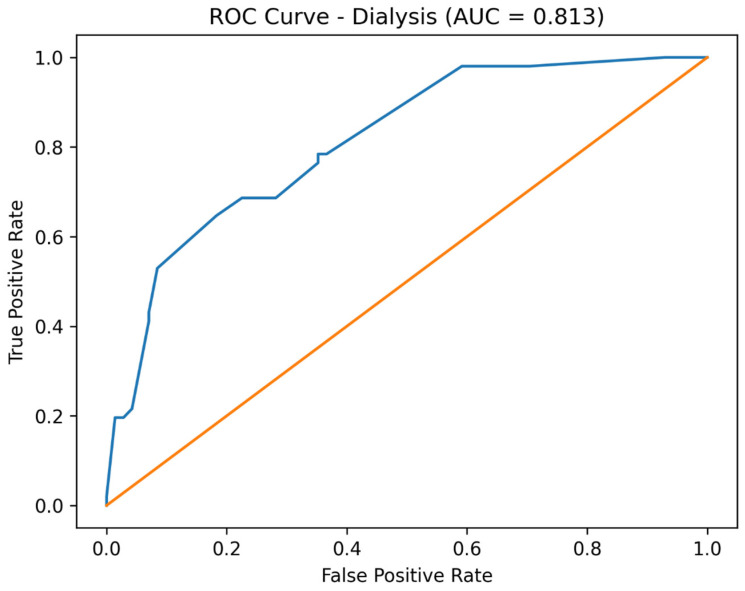
Receiver operating characteristic (ROC) curve for prediction of dialysis using the adjusted logistic regression model (AUC = 0.813). The orange line represents the ROC curve of the adjusted model.

**Figure 3:**
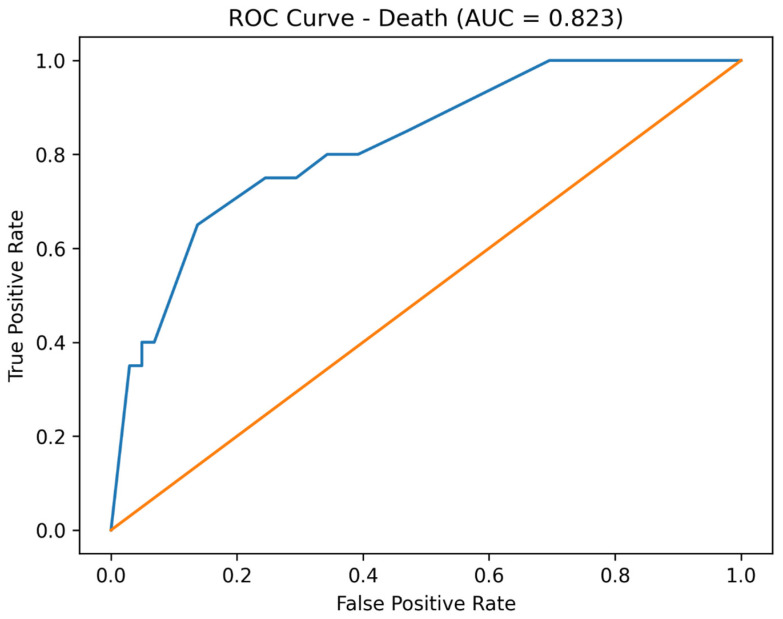
Receiver operating characteristic (ROC) curve for prediction of mortality using the adjusted logistic regression model (AUC = 0.823). The orange line represents the ROC curve of the adjusted model.

Receiver operating characteristic (ROC) curve analysis demonstrated good discriminative performance of the adjusted multivariable logistic regression models. The area under the curve (AUC) was 0.813 for dialysis, 0.838 for amputation, and 0.823 for mortality (Figure 1, Figure 2 and Figure 3).

## 4. Discussion

The present study provides additional evidence supporting the clinical relevance of early targeted anti-rhabdomyolysis infusion therapy in patients with combat-related lower extremity injuries requiring prolonged tourniquet application. Using updated statistical modeling and expanded outcome analysis, we demonstrate that initiation of infusion therapy prior to tourniquet removal is independently associated with reduced odds of dialysis-requiring acute kidney injury, limb amputation, and mortality after adjustment for baseline physiological severity.

Rhabdomyolysis remains a frequent and potentially devastating complication of severe trauma, particularly in military and disaster settings characterized by extensive muscle injury, blast mechanisms, and prolonged ischemia [1,2,3]. In contrast to civilian trauma, combat casualties are exposed to a combination of massive soft-tissue destruction, delayed evacuation, and prolonged tourniquet use, all of which significantly increase the risk of muscle necrosis and systemic toxicity [4,5,6]. Acute kidney injury represents the most clinically significant systemic consequence of rhabdomyolysis and is consistently associated with increased morbidity, mortality, and resource utilization [2,7].

The pathophysiology of rhabdomyolysis-associated AKI is complex and multifactorial. Myoglobin released from damaged skeletal muscle plays a central role through direct tubular toxicity, intratubular cast formation with uromodulin, oxidative injury mediated by free iron, and renal vasoconstriction leading to medullary hypoxia [2,6,7,8,9]. Experimental studies have demonstrated that acidic urinary conditions promote myoglobin precipitation and amplify oxidative stress, thereby exacerbating tubular injury [8]. These mechanisms provide a strong biological rationale for early interventions aimed at optimizing renal perfusion, urine flow, and urinary pH before systemic exposure to myotoxic metabolites.

In the context of prolonged tourniquet application, the ischemic limb initially functions as a relatively isolated compartment, limiting systemic dissemination of myoglobin and other intracellular breakdown products [4,10]. While this effect may transiently protect vital organs, reperfusion following tourniquet release results in an abrupt systemic surge of myoglobin, potassium, lactate, and inflammatory mediators. Clinical and experimental data suggest that ischemia exceeding 3–6 h substantially increases the likelihood of irreversible muscle injury and severe reperfusion-associated toxicity [4,11,12,13].

Consistent with previous observations, overt clinical signs of rhabdomyolysis were often absent prior to tourniquet removal in our cohort. Urinary discoloration and laboratory abnormalities typically appeared only after reperfusion, underscoring the limited value of relying on visible clinical markers to guide preventive interventions [1,2]. This temporal dissociation supports the concept that prophylactic therapy initiated before reperfusion may be more effective than reactive treatment after systemic injury has already occurred.

In unadjusted analyses, differences in adverse outcomes between treatment groups were not uniformly statistically significant. However, after adjustment for baseline shock index and metabolic status, early infusion therapy emerged as an independent protective factor across all studied outcomes. This finding highlights the importance of accounting for physiological severity when evaluating treatment effects in heterogeneous trauma populations. Similar masking of therapeutic effects by baseline instability has been described in other trauma and critical care studies [6,7].

Importantly, model performance improved substantially after adjustment, as demonstrated by marked reductions in AIC values and acceptable-to-good discrimination on ROC analysis. These results suggest that the observed associations are not merely statistical artifacts but reflect meaningful prognostic information captured by the multivariable models.

Across all three outcomes, the magnitude of the association between early infusion therapy and adverse outcomes increased after adjustment for baseline physiological severity. This pattern likely reflects negative confounding, whereby patients who received early anti-rhabdomyolysis infusion therapy presented with greater initial physiological instability, as indicated by higher shock index values.

Adjustment for baseline severity therefore reduced confounding by indication and unmasked a stronger protective association of early infusion therapy. Similar patterns have been reported in observational studies of critically ill and trauma populations, where early interventions are preferentially applied to patients with higher baseline risk.

An important consideration is that the observed association between early infusion therapy and improved outcomes may reflect not only the timing of anti-rhabdomyolysis measures relative to reperfusion, but also more effective early hemodynamic optimization. Patients in the early-treatment group demonstrated lower shock index values prior to tourniquet release, indicating improved circulatory stability before reperfusion.

Nevertheless, the possibility of model instability related to the relatively limited sample size cannot be fully excluded and should be considered when interpreting the magnitude of adjusted effect estimates.

The reduction in dialysis-requiring AKI observed in the early-treatment group is of particular clinical significance. Acute kidney injury not only worsens short-term outcomes but also increases the risk of long-term renal dysfunction and mortality [7,14]. In wartime and disaster settings, where access to renal replacement therapy may be limited or delayed, prevention of severe AKI represents a critical therapeutic objective [5,14]. Our findings support the concept that early renal protective strategies may reduce the burden of dialysis-dependent renal failure in such environments.

The role of bicarbonate and mannitol therapy in rhabdomyolysis remains controversial. While aggressive crystalloid resuscitation is universally recommended as the cornerstone of management [1,9], systematic reviews and meta-analyses have produced inconsistent conclusions regarding the additional benefit of urine alkalization and osmotic diuresis [9,10]. Some authors report no clear advantage beyond early fluid therapy, whereas others emphasize potential benefits in selected high-risk patients with severe ischemia–reperfusion injury [8,15]. The protocol evaluated in the present study does not allow isolation of the individual contribution of each component; however, the observed associations suggest that the combined early approach may be beneficial when initiated before reperfusion.

Current trauma and military guidelines emphasize hemorrhage control and early volume resuscitation but do not uniformly address prophylactic strategies for rhabdomyolysis-associated AKI in the setting of prolonged tourniquet use [15,16]. In this context, the treatment approach evaluated in our study reflects an adaptation driven by frontline clinical experience and pathophysiological reasoning rather than formal guideline recommendations.

In conclusion, this study suggests that in combat-related lower extremity injuries requiring prolonged tourniquet application, early initiation of targeted anti-rhabdomyolysis infusion therapy prior to reperfusion is associated with reduced renal complications, limb loss, and mortality. These findings support further prospective, multicenter investigation to confirm efficacy and to refine optimal preventive strategies in both military and civilian mass-casualty settings.

### Limitations

This study has several limitations. Its retrospective single-center design precludes causal inference and may be subject to residual confounding despite multivariable adjustment and propensity score–based sensitivity analyses. The relatively limited sample size may have affected the precision of effect estimates.

In addition, treatment allocation was not randomized and reflected organizational and temporal differences in clinical practice. The absence of key trauma severity variables, such as Injury Severity Score, detailed injury mechanism, and exact transfusion requirements, further limits causal interpretation of the observed associations. Finally, long-term renal and functional outcomes were not assessed.

Detailed data on transfusion ratios, vasopressor use, and specific damage control resuscitation strategies were not consistently available and therefore could not be included in the analysis.

## 5. Conclusions

In this retrospective cohort study of combat-related lower extremity injuries requiring prolonged tourniquet application, early initiation of an anti-rhabdomyolysis-oriented infusion strategy prior to tourniquet release was associated with lower rates of dialysis-requiring acute kidney injury, limb amputation, and mortality. These associations remained consistent after adjustment for baseline physiological severity and in propensity score–based sensitivity analyses.

Importantly, the observed associations likely reflect the potential benefit of an early, proactive resuscitation approach initiated before reperfusion, rather than a specific isolated pharmacological effect. Improved hemodynamic stabilization prior to reperfusion, along with volume expansion and renal-protective measures, may collectively contribute to the observed outcomes in patients at high risk of ischemia–reperfusion injury and rhabdomyolysis.

Given the retrospective design and the bundled nature of the intervention, the findings should be interpreted as hypothesis-generating. Further prospective, multicenter studies are warranted to determine the independent contribution of infusion timing, hemodynamic optimization, and specific therapeutic components, and to define optimal preventive strategies for rhabdomyolysis-associated complications following prolonged tourniquet application.

## Figures and Tables

**Table 1:** Demographic and clinical characteristics of the study groups.

*Median (QI–QIII)*			
Variable	Standard Treatment	Early Infusion Therapy	*p*-Value
Age; years	33.0 (26.0–35.0)	34.0 (33.0–36.0)	0.143
TAT; hours	4.0 (3.0–6.0)	4.0 (4.0–6.0)	0.754
SI-1	1.1 (1.0–1.1)	1.1 (1.0–1.2)	0.202
SI-2	1.0 (0.8–1.1)	0.8 (0.8–0.9)	**<0.001**
Hb-1; g/L	5.0 (0.0–5.0)	0.0 (0.0–5.0)	0.047
Hb-2; g/L	15.0 (10.0–25.0)	25.0 (15.0–25.0)	**0.027**
Hb-3; g/L	20.0 (10.0–25.0)	25.0 (10.0–25.0)	0.877
pH-1	6.0 (6.0–6.0)	6.0 (6.0–6.0)	0.463
pH-2	7.0 (6.0–7.0)	7.0 (6.0–7.0)	0.086
pH-3	7.0 (7.0–7.0)	7.0 (6.0–7.0)	0.052
Cr-24; µmol/L	445 (332–502)	343 (314–457)	**0.007**

Bold values indicate statistical significance (*p* < 0.05).

**Table 2:** Clinical outcomes.

Outcome	Standard Treatment, *n* (%)	Early Infusion Therapy, *n* (%)
Dialysis	29 (47.5%)	22 (36.1%)
Amputation	16 (26.2%)	12 (19.7%)
Death	13 (21.3%)	7 (11.5%)

**Table 3:** Logistic regression and sensitivity analyses. Bold values indicate statistically significant associations (*p* < 0.05).

Outcome	Model	OR	95% CI	*p*-Value	AIC
Dialysis	Crude	0.62	0.30–1.29	0.200	168.18
Dialysis	Adjusted	**0.33**	**0.13–0.84**	**0.020**	**136.27**
Amputation	Crude	0.69	0.29–1.61	0.390	134.69
Amputation	Adjusted	**0.32**	**0.11–0.95**	**0.040**	**105.45**
Death	Crude	0.48	0.18–1.30	0.148	110.68
Death	Adjusted	**0.23**	**0.07–0.77**	**0.017**	**92.65**

Adjusted models were corrected for shock index at admission and baseline acid–base status. Lower AIC values indicate improved model fit. Sensitivity analysis using propensity score–based inverse probability of treatment weighting yielded comparable effect estimates.

## Data Availability

The data presented in this study are available on reasonable request from the corresponding author.
